# Supplementary material for: COVID-19 vaccine hesitancy and conspiracy beliefs in Togo: Findings from two cross-sectional surveys
Source: PLOS Glob Public Health. 2024 Feb 29;4(2):e0002375. doi: 10.1371/journal.pgph.0002375 (PMC10903826; doi:10.1371/journal.pgph.0002375)
Supplement: S1 Text — (DOCX) [file pgph.0002375.s001.docx]

**Réticence vaccinale contre la COVID-19 et croyances complotistes au Togo : Résultats de deux enquêtes transversales**

Herve Akinocho^1^*; Ken Brackstone^2^*; Nia Eastment^2^*; Jean-Paul Fantognon^3^*; Michael G Head^2,4,5^*

* Afin de démontrer l'équité entre les co-auteurs, ils sont classés par ordre alphabétique des noms de famille

1. Center for Research and Opinion Polls, Lomé, Togo

2. Clinical Informatics Research Unit, Faculty of Medicine, University of Southampton, Southampton, United Kingdom

3. Ministry of Health, of Public Hygiene, and Universal Health Coverage , Lomé, Togo

4. School of Medicine, University for Development Studies, Tamale, Ghana

5. School of Public Health, University for Health and Allied Sciences, Hohoe, Ghana

Auteur contact – Dr Michael Head, [M.Head@soton.ac.uk](mailto:M.Head@soton.ac.uk)

Mots clés – hésitation vaccinale ; désinformation ; infodémie ; COVID-19 ; promotion sanitaire

**Abstract**

**Introduction**

Le Togo est un pays à faible revenu d’Afrique de l’Ouest. En juin 2023, le pays a rapporté 39.503 cas de COVID-19 dont 290 décès confirmés. Selon les estimations, seulement 25% de la population togolaise a reçu au moins une dose d’un quelconque vaccin contre la COVID-19 en juin 2023. Alors que la première phase du déploiement des vaccins en 2021 a été dominé par les pays les plus riches qui ont absorbé une grande partie de l’offre disponible, les approvisionnements sont aujourd’hui suffisants pour toutes les nations. Ainsi, il reste nécessaire de comprendre les raisons de la faible utilisation du vaccin dans les pays comme le Togo, afin d’en tirer les leçons en prévision d’éventuelles urgences de santé publique similaires à l’avenir.

**Méthodes**

Deux enquêtes téléphoniques transversales ont été menées auprès des résidents du Togo en décembre 2020 et janvier 2022. Ces enquêtes posaient des questions sur les perceptions sur la COVID-19, la confiance dans les messages de santé publique, la croyance dans les théories complotistes et l'hésitation autour de la vaccination contre la COVID-19.

**Résultats**

Considérant l'enquête 1 (N = 1430) et l'enquête 2 (N = 212), 65 % des répondants étaient des hommes, 47 % vivaient à Lomé (capitale du Togo), 25 % avaient fait des études supérieures, 67 % étaient mariés et 69 % étaient chrétiens. Entre les enquêtes 1 et 2, la réticence globale au vaccin a considérablement augmenté, passant de 33 % à 58 %. Les croyances dans les théories complotistes ont également augmenté de 29% à 65%. Un modèle de régression logistique a révélé que la méfiance envers le gouvernement était le prédicteur le plus significatif de la réticence vaccinale (OR : 2,90). Le fait d’être d’accord ou incertain avec au moins une théorie complotiste est également un bon prédicteur d’une plus grande réticence vaccinale (OR : 1,36).

**Discussion**

Le taux de réticence parmi les personnes non vaccinées au Togo a augmenté entre décembre 2020 et janvier 2022. Les groupes clés qui étaient plus susceptibles d'exprimer une réticence à la vaccination comprenaient ceux qui avaient une forte méfiance à l'égard du gouvernement et ceux qui croyaient en au moins une théorie complotiste clé autour de la vaccination contre la COVID-19.

Les approches qui peuvent soutenir l’adhésion à l’utilisation des vaccins de la COVID-19 au Togo comprennent des campagnes de promotion sanitaire qui utilisent des personnes ressources de confiance aux niveaux local et national (par exemple, les services de santé ou les chefs religieux) pour une plus grande efficacité. De futures recherches devraient se concentrer sur les gaps de connaissances crées par la pandémie, telles que l'impact de la désinformation sur l’observation du calendrier vaccinal de routine.

**Introduction**

La pandémie de la COVID-19, causée par un nouveau coronavirus qui s’est propagé dans plusieurs pays au début de 2020, était probablement responsable d'environ 18,2 millions de décès supplémentaires dans le monde à la fin de l’année 2021.^1^ La propagation soutenue du virus a eu un impact considérable sur les services de santé de routine à l'échelle mondiale^2^, avec des conséquences socio-économiques énormes et durables. Le Togo est un pays à faible revenu d’Afrique de l’Ouest qui compte environ 8,7 millions d'habitants.Il est limité par le Ghana à l'Ouest, le Bénin à l'Est, le Burkina Faso au Nord et l'Océan Atlantique au Sud. En juin 2023, le pays avait signalé 39 503 cas de COVID-19, avec 290 décès confirmés.^3^ Ces chiffres sont probablement largement sous-estimés, les données montrant que les pays d’Afrique subsaharienne (ASS) ont largement sous-déclaré la maladie, avec un taux de létalité de l’infection ajusté à l’âge probablement plus élevé^4^, ainsi qu'un taux plus élevé de la mortalité chez les patients hospitalisés.^5^ Cependant, il semblerait toujours que la plupart des pays du continent africain n'aient pas connu les taux de mortalité élevés observés dans les pays à revenu élevé comme le Royaume-Uni ou les États-Unis d’Amérique. De nombreux facteurs contribuent probablement à la faiblesse de la mortalité en Afrique de l'Ouest, notamment une population plus jeune, une meilleure préparation^6^ et une meilleure gestion des épidémies.^7^

À la mi-2023, la COVID-19 demeure toujours un fardeau dans le monde, mais elle est plus gérable. La plupart des pays se tournent vers un environnement postpandémique, avec une prise en charge des cas de COVID-19 intégrée dans la routine du système de santé. Cependant, l'utilisation du vaccin contre la COVID-19 au niveau de la population reste faible dans de nombreux pays à faible revenu, avec des estimations selon lesquelles seulement 25 % de la population togolaise a reçu au moins une dose d’un quelconque vaccin contre la COVID-19 (en juin 2023).^3^ Alors que lors des premières mises sur le marché de vaccins tout au long de 2021, ont été dominées par les pays à revenus élevés qui ont absorbé la grande partie des disponibilités,^8^ les approvisionnements sont aujourd’hui suffisants pour toutes les nations. Ainsi, il reste nécessaire de comprendre les raisons de la faible utilisation du vaccin dans les pays comme le Togo afin d’en tirer les leçons en prévision d’une future urgence de santé publique similaire.

La réticence à la vaccination en Afrique de l'Ouest a été associée avec l'insatisfaction et la méfiance envers les gouvernements, en particulier à l'égard des campagnes gouvernementales, par exemple le boycott du vaccin contre la poliomyélite dans le Nord du Nigeria en 2003-2004.^9^ Des enquêtes plus récentes administrées dans des pays d'ASS tels que le Malawi, le Mali et le Nigeria, ont constaté que l'insatisfaction à l'égard de la réponse du gouvernement à la pandémie de COVID-19 prédisait la réticence vaccinale.^10^ Cette tendance a également été constatée au Ghana, où les partisans des partis politiques d'opposition ont manifesté une plus grande hésitation à recevoir le vaccin de la COVID-19 que les partisans du parti actuellement au pouvoir.^11^ Les enquêtes Afrobaromètre ont révélé que même si la proportion de citoyens togolais pensant que leur gouvernement ne puisse garantir l’innocuité des vaccins a diminué entre janvier 2021 et mars 2022, cette dernière reste cependant élevé (62 % en janvier 2021 à 47 % en mars 2022). Mais ils étaient satisfaits de la gestion par le gouvernement de la crise de la COVID-19. Ainsi, les différences individuelles en matière de confiance politique et de méfiance peuvent être associées à la réticence à la vaccination au Togo. Des études ont également montré que la désinformation liée à la COVID-19 est courante en Afrique de l'Ouest. Par exemple, une enquête nationale menée au Ghana a révélé que plus de 50 % des citoyens pensaient, ou exprimaient une incertitude, que la COVID-19 était une arme biologique conçue par le gouvernement chinois, et que le virus avait été conçu spécifiquement pour réduire ou contrôler la population.

Auparavant, notre équipe a publié deux études sur la réticence à la vaccination au Ghana, dont l'une s'est déroulée, par voie électronique, quatre fois,^11^ et une enquête ponctuelle réalisée en personne dans une zone rurale.^12^ À ce jour, il y a eu peu de recherches sur la COVID-19 portant sur le Togo. Cette étude couvre les descriptions de la réticence à la vaccination à partir de deux enquêtes nationales auprès de résidents togolais. Une partie des données de l'enquête 1 a déjà été mise à la disposition des décideurs politiques pour examen et la prise de décision.^13^ Ici, nous donnons un aperçu plus approfondi des deux enquêtes, en cherchant à identifier les caractéristiques associées à la réticence vaccinale à l’aide de variables socioéconomiques et démographiques, et à décrire les connaissances et attitudes à l'égard de la pandémie de COVID-19 au Togo, ainsi que l'impact de celle-ci.

**Méthodes**

**Design, participants, et procédure**

Deux sondages téléphoniques représentatifs à l'échelle nationale ont été administrés. L'enquête 1 a été menée en décembre 2020 - environ 6 mois après la notification du premier cas de COVID-19 au Togo et avant que tout vaccin ne soit disponible dans le pays. L'enquête 2 a été mise en œuvre en janvier 2022, environ 9 mois après le début des campagnes de vaccination à la COVID-19 au Togo.

Avant la collecte des données, une analyse de puissance a été effectuée pour déterminer la taille d'échantillon appropriée. Nous avons supposé un niveau de confiance de 95 % et une marge d'erreur d'environ 3 à 5 %, et avons constaté que l'échantillon nécessaire se situait entre 385 et 1 067 participants à chaque enquête. Nous avons atteint ces tailles d'échantillon dans les deux enquêtes. Cependant, en ne considérant que les participants non vaccinés dans le sondage 2, nous n'avons pas atteint le nombre requis.

**Approbation éthique**

Les participants ont donné leur consentement éclairé avant de participer aux enquêtes. Ils ont reçu des informations verbales sur les participants lors de l'appel téléphonique initial et il leur a aussi été offert l’envoi des informations écrites par e-mail ou message privé. Ils ont ensuite eu le temps de déterminer s'ils souhaitaient participer. Tous les participants ont confirmé qu'ils avaient plus de 18 ans avant de procéder à l'enquête. L'étude a reçu les approbations du Comité de Bioéthique sur la Recherche en Santé au Togo (référence 006/2020/CRBS) et du Comité Ethique de recherche de l’Université de Southampton (référence ERGO : 67300).

**Mesures**

**Hésitation vaccinale.** Dans l'enquête 1, on a demandé aux participants : " Lorsqu'un vaccin contre la COVID-19 devient disponible, souhaitez-vous vous faire vacciner ?" (Oui, Non, je ne sais pas). Dans l'enquête 2, les participants ont d'abord indiqué s'ils avaient déjà reçu des doses du vaccin de la COVID-19. Parmi les participants qui ont indiqué qu'ils avaient reçu aucune dose, on a ensuite demandé aux participants : " Quand un vaccin COVID-19 est disponible pour vous personnellement, le prendrez-vous ?" (Oui, Non, je ne sais pas).

**Méfiance gouvernementale.** Les participants ont ensuite indiqué dans quelle mesure ils étaient d'accord avec l'énoncé : « J'ai confiance dans la réponse du gouvernement togolais à la pandémie de COVID-19 » (1 = fortement en désaccord ; 5 = fortement d'accord ; M = 3,90 ; SD = 1,11). La méfiance a été codée en dichotomisant les réponses des participants (fortement en désaccord, plutôt en désaccord ou indécis).

**Croyances complotistes.** Les participants ont indiqué s'ils croyaient en huit croyances complotistes liées à la COVID-19 enregistrées comme circulant en Afrique subsaharienne.^11^ Ils ont sélectionné « oui » s'ils étaient d'accord avec la croyance, « incertain » s'ils n'étaient pas certains de la croyance, ou « non » s'ils n'étaient pas d'accord avec la croyance (par exemple, « Au meilleur de votre connaissance… [COVID-19] est conçu pour réduire ou contrôler la population ».

**Sources d'information.** On a présenté aux participants plusieurs endroits où ils auraient pu rechercher des informations liées à la COVID-19 et la réponse à la pandémie. Il s'agissait notamment des sources d'information traditionnelles (télévision/radio), du service de santé togolais, des représentants du gouvernement et d'Internet (par exemple, les sites Web d'information, les blogs, Google). Les participants ont sélectionné les sources qu'ils utilisaient généralement pour recevoir des informations sur la COVID-19 et les vaccins.

**Variables démographiques.** Enfin, les participants ont indiqué leur âge (codé en <40 et 40>), leur sexe, leur religion (christianisme, musulman, autre, aucune) et leur état civil (jamais marié, cohabitation sans mariage, marié, séparé mais non divorcé, divorcé, veuf). Les variables socioéconomiques couvraient l'éducation (haute [diplôme universitaire ou supérieur] et basse [secondaire supérieur ou inférieur]) et la région (Lomé, Maritime, Plateaux, Centrale, Kara, Savanes).

**Gestion et analyse des données**

Les données ont été examinées à la recherche d'erreurs, apurées et exportées vers IBM SPSS Statistics 28 pour une analyse plus approfondie. Des statistiques descriptives résument les caractéristiques sociodémographiques des répondants. Les statistiques inférentielles ont été réalisées en trois phases. Premièrement, les tendances temporelles de l'hésitation et de la prévalence dans la population ont été comparées entre chaque enquête. La réticence vaccinale a été codée en dichotomisant les réponses des participants (regroupant non, je ne sais pas) à la question : " Lorsqu'un vaccin contre la COVID-19 devient disponible, souhaitez-vous vous faire vacciner ?" Des tests de Chi-Square χ^2^ ont été effectués pour évaluer les différences catégorielles dans les taux de réticence à la vaccination et les croyances complotistes entre les Enquêtes 1-2. Des analyses descriptives ont également été faites pour résumer les croyances complotistes et les sources autodéclarées d'informations relatives aux vaccins.

Des régressions logistiques bivariées ont permis d’évaluer les relations entre les prédicteurs individuels et la réticence à la vaccination. Une régression logistique combinée a pris en compte tous les prédicteurs dans un seul modèle, fournissant le test le plus strict des associations potentielles avec la réticence à la vaccination. La réticence au vaccin et ses prédicteurs associés ont été remis à l'échelle de 0 ou 1, ce qui a permis une comparaison directe des tailles d'effet.

**Résultats**

*Participants et caractéristiques sociodémographiques*

*Le tableau 1 présente les statistiques descriptives des participants de l'Enquête 1 (N = 1430) et de l'Enquête 2 (N = 212). La majorité des participants aux deux enquêtes étaient des hommes (66,0 %) par rapport aux femmes (34,0 % ; Mage = 35,74 ; SD = 12,25 ; Fourchette = 18-84). La majorité des participants à toutes les enquêtes vivaient à Lomé (47,4%) et dans les Maritimes (18,0%). De plus, 24,7% ont terminé leurs études supérieures contre 75,5% qui ont terminé le deuxième cycle du secondaire ou moins, et 66,8% ont déclaré être mariés ou en couple contre 33,2% qui n'étaient pas en couple. L’analyse par religion montre que 69,2 % des participants étaient chrétiens par rapport aux musulmans ou autres/aucun (16,3 % et 14,5 %, respectivement). Enfin, 72,8 % ont déclaré avoir des responsabilités de soins pour des moins de 18 ans ou des adultes plus âgés.*

*Sources d'informations relatives au vaccin COVID-19*

*Les sources d'information sur le vaccin de la COVID-19 les plus consultées étaient les médias (journaux, radio, télévision ; 94,4 %), les services de santé togolais (58,2 %), les représentants du gouvernement (33,4 %), et l’Internet (par exemple, Google, sites d’information, blogs ; 25,6 %)*

Croyances complotistes

Dans l'ensemble, 33,7 % (554/1088) des participants ont indiqué être d'accord avec au moins une croyance complotiste (M = 0,57, SD = 1,03 ; Tableau 2). Les croyances complotistes les plus communément admises étaient : « … un fléau causé par les péchés et l'incrédulité des êtres humains » (261/1642 ; 15,9 %), « … une arme biologique conçue par le gouvernement chinois » (157/1642 ; 9,6 %), et « … conçu pour réduire ou contrôler la population » (151/1642 ; 9,2 %). Un test du chi carré de Pearson a révélé une association significative entre le temps et les croyances complotistes, dans laquelle la proportion de répondants qui ont indiqué être d’accord est passée de 29,2 % (IC : 26,6 % -31,8 %) dans l'enquête 1 (décembre 2020) à 64,6 % (IC à 95 % : 57,9 %-71,3 %) dans l'enquête 2 (janvier 2022 ; χ^2^ (1) = 103,85, p < 0,001 ; Figure 1).

Ensuite, 48,4 % (794/1642) des participants ont indiqué une incertitude concernant au moins une croyance complotiste liée à la COVID-19 (M = 2,42, SD = 2,99). Les croyances complotistes les plus courantes incluaient : « … un virus conçu par l'industrie pharmaceutique pour vendre ses médicaments » (601/1642 ; 36,6 %), « … est une arme biologique causée par le gouvernement américain » (572/1642 ; 34,8 %), et « … conçu pour réduire ou contrôler la population » (555/1642 ; 33,8 %). Un test du chi carré de Pearson a révélé une association significative entre le temps et les croyances complotistes, dans laquelle la proportion de répondants est passée de 43,5 % (IC : 40,9 %-46,1 %) dans l'enquête 1 (décembre 2020) à 81,1 % (95 % IC : 74,4 %-87,8 %) dans l'enquête 2 (janvier 2022 ; χ^2^ (1) = 104,72, p < 0,001 ; Figure 1).

Réticence à la vaccination

Un test du chi carré de Pearson a révélé une association significative (Figure 2) entre le temps et la réticence à la vaccination (χ^2^ (1) = 52,23, p < 0,001), dans laquelle la réticence globale est passée de 32,5 % (IC : 29,9 % à 35,1 %) dans l'enquête 1 (décembre 2020) à 58,0 % (IC à 95 % : 51,3 %-64,7 %) dans l'enquête 2 (janvier 2022).

Le tableau 3 montre le modèle de régression logistique combinant les facteurs contribuant à la réticence à la vaccination contre la COVID-19. La méfiance gouvernementale était le prédicteur le plus puissant de la réticence dans le modèle (OR : 2,90 ; IC à 95 % : 2,23-3,79 ; p < 0,001) (Figure 3). De plus, les participants qui ont indiqué un accord ou une incertitude avec au moins une croyance complotiste (c'est-à-dire les participants qui ont coché « oui » pour indiquer un accord ou « Je ne sais pas » pour indiquer une incertitude) ont prédit une plus grande réticence à la vaccination par rapport aux participants qui n'ont pas indiqué d'accord ou incertitude (OR : 1,36 ; IC à 95 % : 1,07-1,72 ; p = 0,010).

Il n'y avait pas de prédicteurs significatifs de la réticence à la vaccination parmi les participants qui ont utilisé le Service de santé du Togo (OR : 1,15 ; IC à 95 % : 0,91-1,45 ; p = 0,25), les médias (OR : 0,74 ; IC à 95 % : 0,47-1,16 ; p = 0,189), ou des responsables gouvernementaux (OR : 0,81 ; IC à 95 % : 0,64-1,04 ; p = 0,095) pour obtenir les informations relatives au vaccin COVID-19 par rapport aux participants qui ont déclaré ne pas utiliser ces plateformes. Cependant, les participants qui ont déclaré utiliser des pages Web Internet (p. : 1,06-1,82 ; p = 0,016). Cependant, les participants qui ont déclaré utiliser des pages Web Internet (par exemple, sites d’information, blogs, Google) comme source d’informations relative à la COVID-19 ont significativement une plus grande chance de signaler une réticence vaccinale plus que ceux n’utilisant pas Internet (OR: 1.39; 95% CI: 1.06-1.82; *p* = .016).

Enfin, il y avait plusieurs facteurs démographiques et sociodémographiques importants. Une plus grande réticence a été observée chez les participants musulmans par rapport aux participants chrétiens (OR : 1,33 ; IC à 95 % : 1,05-1,69 ; p = 0,018) et une réticence significativement plus faible chez les participants vivant dans la Kara (OR : 0,58 ; IC à 95 % : 0,38 -0,90 ; p = 0,015) et les savanes (OR : 0,45 ; IC 95 % : 0,26-0,76 ; p = 0,003) du Togo par rapport à Lomé. Il y avait aussi une réticence légèrement plus élevée chez les femmes que chez les hommes (OR : 1,23 ; IC à 95 % : 0,97-1,53 ; p = 0,080).

**Discussion**

Cette étude décrit les preuves de changements dans les niveaux globaux de réticence à la vaccination au Togo à deux moments dans le temps durant la réponse à la pandémie de COVID-19. La réticence a augmenté entre décembre 2020 et janvier 2022. L'enquête 2 a eu lieu après la mise à disposition des vaccins de COVID-19 au Togo, avec les campagnes de promotion associées qui ont accompagné la mise à disposition du vaccin. La croyance ou l'incertitude autour des principales théories du complot a également augmenté entre les enquêtes 1 et 2. Parmi les groupes clés les plus susceptibles d'exprimer une réticence, on trouve les musulmans, les résidents, les électeurs des partis d'opposition, les femmes, les personnes qui ont reçu des informations sur le COVID-19 à partir de sources Internet et les personnes qui ont exprimé leur incertitude concernant les croyances de désinformation COVID-19 couramment diffusées.

Nos résultats montrent ici que la présence de théories du complot peut avoir un impact sur la perception publique de la vaccination. Il n'y a pas de technologie 5G mise en place au Togo - malgré cela, environ un tiers des participants ici ont indiqué qu'ils ne savaient pas si la 5G était responsable de la propagation de la COVID-19. Pendant la pandémie, le terme « infodémie » a été utilisé pour décrire la prévalence à la fois de trop d'informations et de fausses informations, ce qui a conduit, par exemple, la région Afrique de l'OMS à mettre en place une alliance pour la réponse à l'infodémie.^14^ La portée de cette alliance est de surveiller et contrer de manière proactive la désinformation, les théories du complot et les rumeurs, soutenant ainsi les acteurs des services de santé dans leur engagement auprès du grand public. L'urgence de santé publique de la variole du singe a également été marquée par une « infodémie », avec des quantités importantes de désinformation.^15^ Cela peut avoir un impact négatif sur toute réponse des systèmes de santé, les individus se sentant stigmatisés et réticents à se faire soigner.

La confiance dans la gouvernance est apparue comme un facteur important concernant les messages de santé publique et le contrôle des infections pendant la pandémie de COVID-19.^16^ Nos résultats du Ghana ont montré que l'allégeance politique a un rôle à jouer dans la confiance autour de la vaccination.^11^ Lorsque le gouvernement est la source de tout message de promotion de la santé, cela peut être plus volontiers reçu par les populations qui ont voté pour eux. Les électeurs de l'opposition peuvent faire moins confiance à ces messages et, par conséquent, nécessitent une attention particulière quant à savoir qui communique les messages de santé publique et comment ils peuvent avoir le meilleur impact possible. Au-delà de la pandémie, la confiance dans les vaccins du calendrier vaccinal de routine devra être surveillée et de nouvelles voies explorées pour contrer toute désinformation.

Une année après leur utilisation, on estime que les vaccins de COVID-19 ont évité entre 15 et 20 millions de décès dans le monde.^17^ Pour le Togo, en 2020 et 2021, il y a eu 248 décès confirmés. Cependant, la modélisation estime le nombre de décès excédentaires à environ 9030, soit une différence de ratio de 36,4 entre la mortalité confirmée et la surmortalité.^1^ Il y aura sans aucun doute un nombre important de décès dus à la COVID-19 qui ne seront jamais reconnus dans les statistiques officielles. Un manque d'infrastructures pour collecter des données de routine, y compris les registres de naissances et de décès, signifie que de nombreuses personnes dans les pays à faible revenu, y compris le Togo, ne figurent jamais dans les statistiques officielles.^18^ Compte tenu de ces limites, il est très difficile de fournir des informations précises en temps réel lors d'une urgence de santé publique, telle que la pandémie de COVID-19. Avant la prochaine pandémie ou épidémie très médiatisée, la mise en œuvre de systèmes de collecte de données de routine améliorés permettrait d’élaborer une politique et une planification mieux informées.

L'un des points forts de notre étude est le nombre relativement important de répondants et le fait qu'elle couvre deux périodes dans le temps, plutôt qu'un cliché unique de l'opinion publique. Ces points de temps couvraient également avant et après l'introduction des vaccins de COVID-19. Cependant, la nécessité d'un accès à Internet ou au téléphone peut avoir limité dans une certaine mesure la représentativité de la population échantillonnée. Certains groupes seront sous-représentés, y compris ceux qui résident dans les zones rurales et les personnes de statut socio-économique inférieur - les populations dites du dernier kilomètre.^19^ Il y aura probablement un certain biais de réponse, étant donné que les répondants étaient tenus de donner leur consentement et de participer via une sélection aléatoire à partir d'un appel téléphonique ou avoir vu l'enquête sur Internet et choisi d’y répondre. Cependant, au moment où l'enquête a été achevée, des directives nationales étaient en place concernant les mouvements de population, et les approches à distance étaient donc plus réalisables, acceptables localement et plus rentables que la collecte de données en personne dans des communautés spécifiques. Nous avons également pu obtenir des réponses à l'échelle nationale grâce à ces méthodes, ce qui aurait peut-être été moins faisable avec des agents de collecte de données sur le terrain.

**Conclusion**

Les taux réticence parmi les personnes non vaccinées au Togo ont augmenté entre décembre 2020 et janvier 2022. Parmi les groupes clés les plus susceptibles d'exprimer une réticence, il y avait ceux qui avaient une forte méfiance à l'égard du gouvernement et croyaient en au moins une théorie du complot clé autour de la vaccination contre la COVID-19. Les campagnes de promotion de la santé doivent faire appel à des personnes ressources de confiance aux niveaux local et national (par exemple, les services de santé) et diffuser les messages de santé publique via des personnes de confiance, telles que des chefs religieux. Les messages doivent également cibler les plateformes médiatiques utilisées par les groupes de population hésitants. Il doit y avoir une prise de conscience de la portée et de la force de la désinformation en circulation, et adopter des approches proactives de promotion de la santé pour contrer cette désinformation. Par exemple, les campagnes peuvent se concentrer sur les préoccupations concernant la sécurité des vaccins et les effets secondaires. Ces approches peuvent améliorer l'adhésion des populations à l’utilisation de tous les vaccins de COVID-19 au Togo. Les futures recherches devraient se concentrer sur les gaps dans les connaissances créés par la pandémie, telles que l'impact de la désinformation sur le recours au calendrier vaccinal de routine.

**Financement**

Le financement de cette étude a été mise à disposition par “the Clinical Informatics Research Unit, at the University of Southampton”.

**Remerciements**

Nous reconnaissons et remercions les participants à l'enquête et les agents de collecte de données pour leur temps et leur aide dans cette recherche.

**Conflits d’intérêts**

Aucun auteur n'a de conflits d'intérêts à déclarer.

**Contribution des auteurs**

La conception et le contenu de l'étude ont été éclairés par les contributions de tous les auteurs. MGH a dirigé la rédaction du premier draft de ce projet. KB a effectué les analyses, soutenue par NE. HA a dirigé la collecte des données. JPF a fourni une supervision experte. Tous les auteurs ont examiné et commenté le draft de ce manuscrit. Tous les auteurs ont approuvé la version finale de ce manuscrit.

**References**

1 Wang H, Paulson KR, Pease SA, *et al.* Estimating excess mortality due to the COVID-19 pandemic: a systematic analysis of COVID-19-related mortality, 2020-21. *Lancet* 2022; **399**: 1513–36.

2 Organization WH. Pulse survey on continuity of essential health services during the COVID-19 pandemic: interim report. 2020. https://www.who.int/publications/i/item/WHO-2019-nCoV-EHS_continuity-survey-2020.1.

3 Our World in Data. Togo: Coronavirus Pandemic Country Profile. https://ourworldindata.org/coronavirus/country/togo (accessed June 29, 2023).

4 Levin AT, Owusu-Boaitey N, Pugh S, *et al.* Assessing the burden of COVID-19 in developing countries: systematic review, meta-analysis and public policy implications. *BMJ Glob Health* 2022; **7**. DOI:10.1136/bmjgh-2022-008477.

5 Kirenga BJ, Byakika-Kibwika P. Excess COVID-19 mortality among critically ill patients in Africa. *The Lancet* 2021; **397**: 1860–1.

6 Ahanhanzo C, Johnson EAK, Eboreime EA, *et al.* COVID-19 in West Africa: Regional resource mobilisation and allocation in the first year of the pandemic. *BMJ Glob Health* 2021; **6**: 4762.

7 Danquah EPB, Darko S, Frimpong A, Head MG, Osei B. Lessons from the field: COVID-19 outbreak investigations in Kpone-Katamanso, Greater Accra, Ghana: A Global South approach to disease control and contact tracing. *Trans R Soc Trop Med Hyg* 2022; **116**. DOI:10.1093/trstmh/trac063.

8 Yamey G. Rich countries should tithe their vaccines. *Nature* 2021; **590**: 529.

9 Jegede AS. What Led to the Nigerian Boycott of the Polio Vaccination Campaign? *PLoS Med* 2007; **4**: e73.

10 Kanyanda S, Markhof Y, Wollburg P, Zezza A. Acceptance of COVID-19 vaccines in sub-Saharan Africa: evidence from six national phone surveys. *BMJ Open* 2021; **11**: e055159.

11 Brackstone K, Atengble K, Head M, Boateng L. COVID-19 vaccine hesitancy trends in Ghana: a cross-sectional study exploring the roles of political allegiance, misinformation beliefs, and sociodemographic factors. *Pan Afr Med J* 2022; **43**. DOI:10.11604/pamj.2022.43.165.37314.

12 Afreh OK, Angwaawie P, Attivor EJK, *et al.* Examining confidence and hesitancy towards COVID-19 vaccines: A cross-sectional survey using in-person data collection in rural Ghana. *Vaccine* 2023; **41**. DOI:10.1016/j.vaccine.2023.02.024.

13 Head M, Brackstone K, Akinocho H, *et al.* Examining determinants of COVID-19 vaccine hesitancy in Togo. 2021; published online June 25. DOI:10.6084/M9.FIGSHARE.14805765.V1.

14 World Health Organization. Africa Infodemic Response Alliance. https://www.afro.who.int/aira (accessed July 14, 2023).

15 Farahat RA, Head MG, Tharwat S, *et al.* Infodemic and the fear of monkeypox: call for action. *Trop Med Health* 2022; **50**. DOI:10.1186/s41182-022-00459-8.

16 Thornton J. Covid-19: Trust in government and other people linked with lower infection rate and higher vaccination uptake. *BMJ* 2022; **376**: o292.

17 Watson OJ, Barnsley G, Toor J, Hogan AB, Winskill P, Ghani AC. Global impact of the first year of COVID-19 vaccination: a mathematical modelling study. *Lancet Infect Dis* 2022; **0**. DOI:10.1016/S1473-3099(22)00320-6/ATTACHMENT/CF5CA979-59F2-496B-81AB-FB356D7881BD/MMC1.PDF.

18 Hoxha K, Hung YW, Irwin BR, Grépin KA. Understanding the challenges associated with the use of data from routine health information systems in low- and middle-income countries: A systematic review. *Health Information Management Journal* 2022; **51**: 135–48.

19 Pedrajas M, Choritz S. Getting to the Last Mile in Least Developed Countries. 2016. https://www.uncdf.org/article/939/getting-to-the-last-mile-in-least-developed-countries-migration.

**Tableau 1 :** Statistiques descriptives des participants aux deux enquêtes. Les cellules vides indiquent des variables non collectées.

|  | **Combiné (N = 1642)** | **2020 (N = 1430)** | **2022 (N = 212)** |
| --- | --- | --- | --- |
|  | **% (n)** | | |
| **Sex** |  |  |  |
| Homme | 66.0 (1084) | 66.0 (944) | 66.0 (140) |
| Femme | 34.0 (558) | 34.0 (486) | 34.0 (72) |
| **Age** |  |  |  |
| < 40 | 66.2 (1087) | 64.5 (922) | 77.8 (165) |
| 40 > | 33.8 (555) | 35.5 (508) | 22.2 (47) |
| **Statut marital** |  |  |  |
| Célibataire/Divorcé | 33.2 (544) | 31.1 (444) | 47.2 (100) |
| Marié/en relation | 66.8 (1097) | 68.9 (985) | 52.8 (112) |
| **Région** |  |  |  |
| Centrale | 7.9 (129) | 7.6 (109) | 9.4 (20) |
| Kara | 8.3 (136) | 8.8 (126) | 4.7 (10) |
| Lomé | 47.4 (778) | 49.2 (704) | 34.9 (74) |
| Maritime | 18.0 (295) | 16.1 (230) | 30.7 (65) |
| Plateaux | 12.7 (209) | 12.3 (176) | 15.6 (33) |
| Savanes | 5.8 (95) | 5.9 (85) | 4.7 (10) |
| **Niveau le plus élevé d’éducation** |  |  |  |
| Enseignement secondaire ou moins | 75.5 (1237) | 75.9 (1085) | 71.7 (152) |
| Enseignement supérieur | 24.7 (405) | 24.1 (345) | 28.3 (60) |
| **Religion** |  |  |  |
| Chrétienne | 69.2 (1135) | 70.0 (999) | 64.2 (136) |
| Musulmanne | 16.3 (267) | 16.2 (231) | 17.0 (36) |
| Autre ou aucune | 14.5 (237) | 13.8 (197) | 18.9 (40) |
| **Responsable de soins** |  |  |  |
| Non | 27.2 (446) | 24.4 (348) | 46.2 (98) |
| Oui | 72.8 (1194) | 75.6 (1080) | 53.8 (114) |
| **A été vacciné ?** |  |  |  |
| Non | 26.8 (436) | 25.7 (368) | 34.2 (68) |
| Ne sait pas | 8.6 (140) | 6.4 (91) | 24.1 (48) |
| Oui | 64.6 (1053) | 67.8 (970) | 41.7 (83) |
| **Sources d’information** |  |  |  |
| Masse média (TV/radio) | 94.4 (1550) | 97.4 (1393) | 74.1 (157) |
| Travailleurs du domaine de la santé | 58.2 (955) | 63.6 (909) | 21.7 (46) |
| Officiels du gouvernement | 33.4 (549) | 37.0 (529) | 9.4 (20) |
| Internet | 25.6 (421) | 26.7 (382) | 18.4 (39) |

**Tableau 2.** Répartition des croyances en matière de désinformation sur le COVID-19 dans les enquêtes 1 et 2. Remarque : Les pourcentages peuvent ne pas être égaux à 100 en raison de questions incomplètes

|  | **Combiné (N = 1642)** | **2020 (N = 1430)** | **2022 (N = 212)** |
| --- | --- | --- | --- |
|  | **% (n)** | | |
| **Une arme biologique conçue par le gouvernement chinois** |  |  |  |
| Oui | 9.6 (157) | 8.2 (117) | 18.9 (40) |
| Non | 59.0 (969) | 59.6 (852) | 55.2 (117) |
| Je ne sais pas | 31.4 (515) | 32.2 (460) | 25.9 (55) |
| **Un virus conçu par les laboratoires pharmaceutiques pour vendre leurs médicaments** |  |  |  |
| Oui | 5.4 (89) | 2.9 (41) | 22.6 (48) |
| Non | 58.0 (951) | 62.7 (896) | 25.9 (55) |
| Je ne sais pas | 36.6 (601) | 34.4 (492) | 51.4 (109) |
| **Une exagération par les médias d'information pour semer la peur et la panique** |  |  |  |
| Oui | 8.9 (146) | 7.7 (110) | 17.0 (36) |
| Non | 64.7 (1061) | 68.9 (984) | 36.3 (77) |
| Je ne sais pas | 26.4 (434) | 23.4 (335) | 46.7 (99) |
| **Un fléau causé par les péchés et l'incrédulité des êtres humains** |  |  |  |
| Oui | 15.9 (261) | 15.9 (227) | 16.0 (34) |
| Non | 61.9 (1016) | 63.4 (907) | 51.4 (109) |
| Je ne sais pas | 22.5 (365) | 20.7 (296) | 32.5 (69) |
| **Conçu pour réduire ou contrôler la population** |  |  |  |
| Oui | 9.2 (151) | 8.3 (118) | 15.6 (33) |
| Non | 57.0 (935) | 57.7 (824) | 52.4 (111) |
| Je ne sais pas | 33.3 (555) | 34.1 (487) | 32.1 (68) |
| **Une arme biologique conçue par le gouvernement américain** |  |  |  |
| Oui | 5.6 (92) | 1.3 (18) | 34.9 (74) |
| Non | 59.5 (977) | 63.1 (901) | 35.8 (76) |
| Je ne sais pas | 34.9 (572) | 35.7 (510) | 29.2 (62) |
| **Résultat de l'installation de la technologie 5G dans le pays** |  |  |  |
| Oui | 2.4 (40) | 1.1 (16) | 11.3 (24) |
| Non | 65.5 (1075) | 68.2 (974) | 47.6 (101) |
| Je ne sais pas | 32.1 (526) | 30.7 (439) | 41.0 (87) |
| **Transmis par ondes radio** |  |  |  |
| Oui | 0.5 (8) | 0.1 (2) | 2.8 (6) |
| Non | 74.8 (1227) | 78.1 (1116) | 52.4 (111) |
| Je ne sais pas | 24.7 (406) | 21.8 (311) | 44.8 (95) |

**Tableau 3.** Modèle de régression logistique combinant les facteurs contribuant à la réticence à la vaccination contre la COVID-19. *N* = 1642. *R²* = 0.110.

|  | **OR** | **p-value** | **95% CI** |
| --- | --- | --- | --- |
| Age 40+ (ref. = 18-39) | 1.097 | .446 | 0.865 – 1.390 |
| Femme (ref. = Homme) | 1.225 | .080 | 0.976 – 1.538 |
| Région (ref. = Lomé)  Centrale  Kara  Maritime  Plateaux  Savanes | 0.709  0.582  1.130  0.955  0.450 | .118  .015  .416  .788  .003 | 0.461 – 1.091  0.377 – 0.899  0.842 –1.516  0.681 – 1.339  0.264 – 0.765 |
| Enseignement supérieur (ref. = Enseignement secondaire ou moins) | 0.866 | .285 | 0.665 – 1.128 |
| Musulman et autres (ref. = Chrétiens) | 1.333 | .018 | 1.051 – 1.689 |
| Méfiance envers le gouvernement (ref. = Faible méfiance) | 2.904 | < .001 | 2.227 – 3.788 |
| Responsabilités de garde d'adultes ou d'enfants (ref. = aucune) | 0.880 | .302 | 0.689 – 1.122 |
| Croyances complotistes ou incertitude (ref. = aucune) | 1.359 | .010 | 1.074 – 1.719 |
| Sources d'information de la COVID-19  Masse média (exemple radio, journaux, TV)  Service de santé togolais ou agents de santé  Officiels du gouvernement  Internet (exemple, Google, sites d’information, blogs) | 0.738  1.145  0.812  1.390 | .189  .254  .095  .016 | 0.468 – 1.162  0.907 – 1.446  0.636 – 1.037  1.063 – 1.818 |


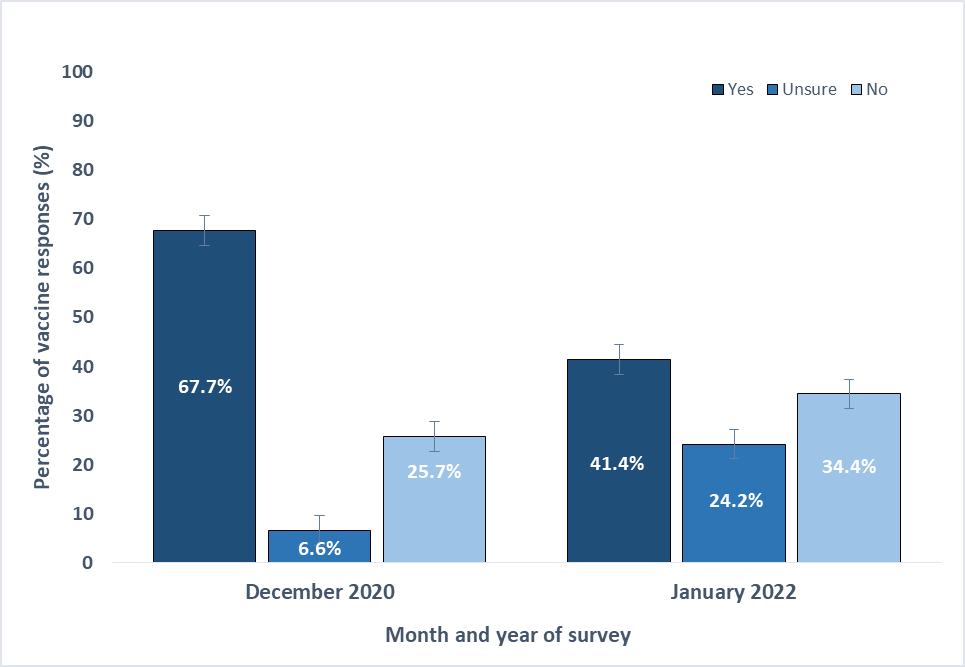


**Figure 2 :** Répartition des réponses oui, non et incertain à travers deux enquêtes représentatives au niveau national menées au Togo en décembre 2020 et janvier 2022.


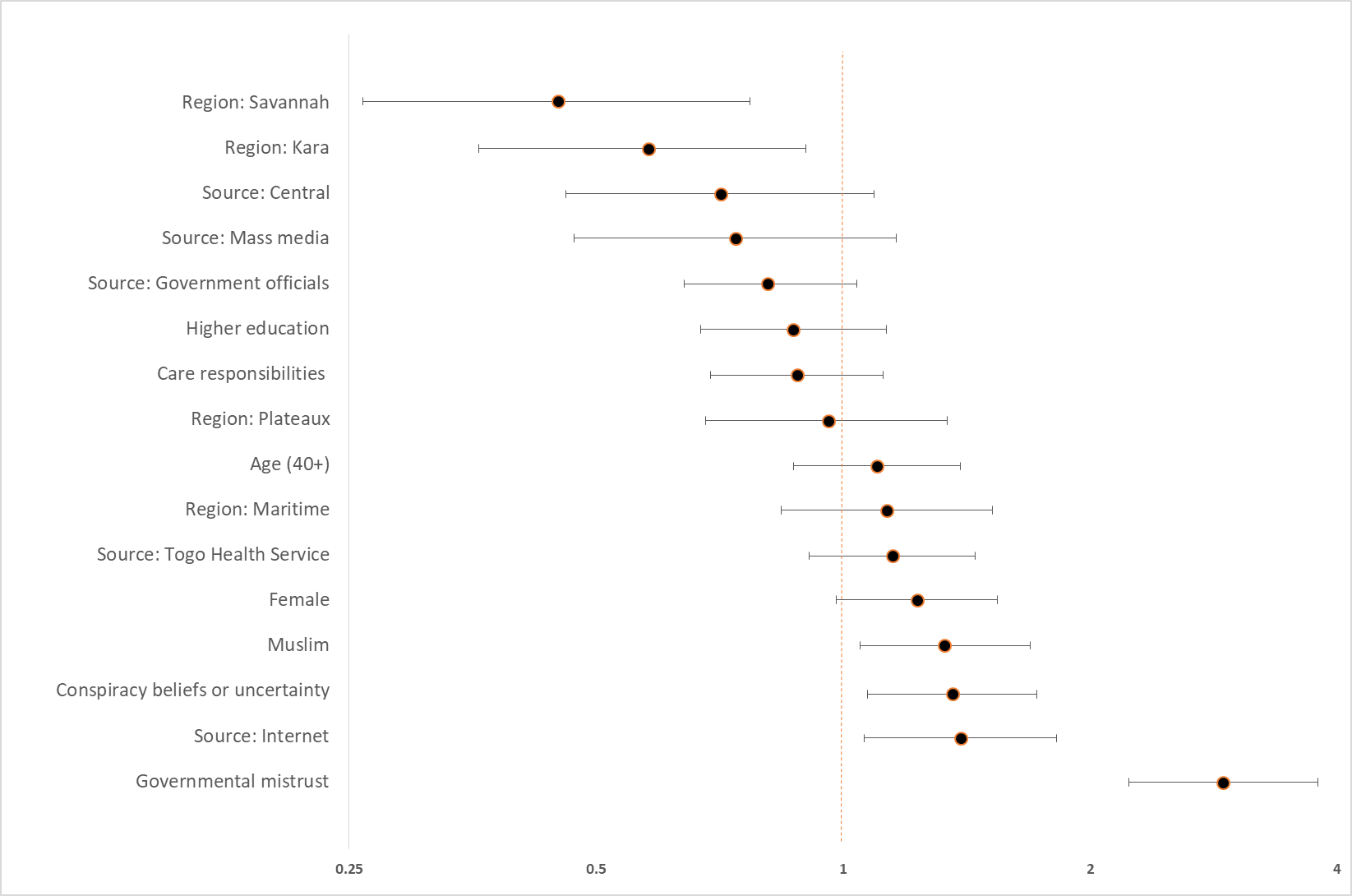


**Figure 3 :** Modèle de régression logistique combinant les facteurs contribuant à la réticence à la vaccination contre la COVID-19 (N = 1138, R2 = 0.144)
