# Supplementary material for: COVID-19 vaccine hesitancy and conspiracy beliefs in Togo: Findings from two cross-sectional surveys
Source: PLOS Glob Public Health. 2024 Feb 29;4(2):e0002375. doi: 10.1371/journal.pgph.0002375 (PMC10903826; doi:10.1371/journal.pgph.0002375)
Supplement: S2 Text — (DOCX) [file pgph.0002375.s002.docx]

**About You**

Q1 **Quel est ton sexe?**

- Homme (1)
- Femelle (2)

Q2 **Quel âge avez-vous?**

________________________________________________________________

Q3 **Dans quelle région habitez-vous actuellement?**

- Centrale (1)
- Kara (2)
- Lomé (3)
- Maritime (4)
- Plateaux (5)
- Savanes (6)

Q4A **Dans quelle préfecture résidez-vous actuellement ?**

Q4B **Dans quel canton/mairie résidez-vous actuellement ?**

Q4C **Dans quel quartier habitez-vous actuellement?**

________________________________________________________________

Q5 **Vivez-vous dans une zone plus rurale ou urbaine (Urbaine = chef lieu de préfecture) ?**

- Rural (1)
- Urbain (2)
- De banlieue (3)
- Je ne sais pas (4)

Q6 **Quelle est votre situation matrimoniale?**

- Célibataire et jamais marié (1)
- En couple mais pas encore marié (ou concubinage) (2)
- Marié/e (3)
- Séparé/e ou divorcé/e (4)
- Veuf/ve (5)

Q7a **Quelle est votre religion?**

- Christianisme (1)
- Islam (2)
- Traditionnel (3)
- Autre (4)
- Sans religion (5)

Q7b **Á quel point êtes-vous religieux ?**

- Pas du tout religieux (1)
- Un peu religieux (2)
- Modérément religieux (3)
- Trés religieux (4)
- Extrément religieux (5)

Q8 **Quel est votre plus haut niveau d'éducation?**

- Pas d'éducation formelle (1)
- École primaire (2)
- Premier cycle du secondaire (3)
- Second cycle du secondaire ou professionnel (4)
- Enseignement supérieur (premier ou troisième cycle) (5)

Q9a **Quelle est votre situation d'emploi actuelle?**

- Sans emploi (1)
- Travailleur indépendant (2)
- Un employé rémunéré (à temps partiel) (3)
- Un employé rémunéré (à temps plein) (4)
- Bourse d'études ou d'apprentissage (5)

Display This Question:

If = Travailleur indépendant (2)

Or = Un employé rémunéré (à temps partiel) (3)

Or = Un employé rémunéré (à temps plein) (4)

Q9b **Quel est votre rôle / titre professionnel?**

________________________________________________________________

Q10 **Dans quelle mesure l'épidémie de COVID-19 a-t-elle *eu un* impact négatif sur votre situation financière au cours des 6 derniers mois ?**

- Pas du tout (1)
- Un peu (2)
- De façon modérée (3)
- Beaucoup (4)
- Enormément (5)

Q11 **Compte tenu de vos propres revenus et des autres revenus de votre ménage, comment décririez-vous votre situation financière dans son ensemble? Diriez-vous que :**

- Vos besoins de base sont non satisfaits (1)
- Les besoins de base sont justes satisfaits (2)
- Besoins de base satisfaits avec un peu de réserve (3)
- Vivez confortablement (4)

Q.12 **Avez-vous des enfants de moins de 18 ans ?**

- Oui (1)
- Non (2)

Q13a **Combien de personnes vivent dans votre ménage au total ?**

________________________________________________________________

Q13b **Combien d'enfants de moins de 18 ans vivent dans votre ménage ?**

________________________________________________________________

Q13c **Combien d'adultes de plus de 60 ans vivent dans votre ménage ?**

________________________________________________________________

**You and COVID-19**

Q14 **Avez-vous déjà été testé pour COVID-19, et si oui, quel a été le résultat du test ?**

- Je n'ai jamais été testé pour COVID-19 (1)
- J'ai déjà été testé et tous les tests étaient négatifs (2)
- J'ai déjà été testé et un ou plusieurs de ces tests étaient positifs (3)

Q15 **En pensant à vos autres membres de votre foyer, l'un d'entre eux a-t-il déjà été testé pour COVID-19 ?**

- Personne d'autre dans ce foyer n'a jamais été testé pour COVID-19 (1)
- Oui, d'autres membres de ma famille ont été testés et tous les tests étaient négatifs (2)
- Oui, d'autres membres de ma famille ont été testés et le test d'au moins une personne était positif (3)

Q16 **En dehors de votre foyer, mais au sein de votre communauté locale (par exemple, ce village ou quartier de ville, votre groupe de d’amis), connaissez-vous personnellement quelqu'un qui a reçu un diagnostic positif de COVID-19 ?**

- Non, je ne connais personne qui a été testé positif pour COVID-19 (1)
- Oui, juste une ou deux personnes (2)
- Oui, plusieurs personnes (par exemple, trois ou plus) (3)

Q17 **Si jamais vous éprouvez des symptômes de la COVID-19 à l'avenir, feriez-vous un test ?**

- Oui (1)
- Non (2)
- Je ne sais pas (3)

**Vaccines**

Q18 **Avez-vous reçu des doses du vaccin COVID-19 ?**

- Non, je n'ai reçu aucune dose (1)
- Oui, j'ai reçu une dose du vaccin COVID-19 (2)
- Oui, j'ai reçu deux doses du vaccin COVID-19 (3)

Display This Question:

If Q18 = (2) or (3)

Q19 **Si vous avez reçu une ou plusieurs doses d'un vaccin COVID-19, savez-vous de quel vaccin il s'agissait ?**

- Pfizer
- Moderna
- Oxford AstraZeneca
- Johnson et Johnson
- Sinovac
- Pas certain

Display This Question:

If Q18 = (2) or (3)

Q20 **Avez-vous ressenti des effets secondaires à la suite de votre première ou de votre deuxième dose de vaccin ?**

- Oui - ma 1ère dose uniquement (1)
- Oui - ma 2ème dose uniquement (2)
- Oui - les deux doses (3)
- Je n'ai ressenti aucun effet secondaire à l'une ou l'autre des doses (4)
- Pas certain (5)

Display This Question:

If Q20 = (1), (2), or (3)

Q21 **Quel (s) effet (s) secondaire (s) avez-vous ressenti de votre (vos) dose (s)?**

________________________________________________________________

Display This Question:

If Q20 = (1), (2), or (3)

Q22 **Quelles mesures avez-vous prises pour gérer les effets secondaires de vos doses (le cas échéant?)** Veuillez cocher tout ce qui s'applique.

- Je n'ai rien fait pour les effets secondaires (1)
- J'ai pris des médicaments pour contrôler ces effets secondaires (2)
- J'ai rendu visite à un professionnel de la santé pour obtenir des conseils sur les effets secondaires (3)
- J'ai pris un congé en raison des effets secondaires (4)
- J'ai annulé les engagements sociaux en raison des effets secondaires (5)
- J'ai demandé des conseils sur les effets secondaires sur Internet (6)
- J'ai demandé des conseils sur les effets secondaires des médias sociaux (7)
- J'ai demandé des conseils sur les effets secondaires à des membres de la famille ou à des amis (8)
- J'ai été admis à l'hôpital en raison des effets secondaires (9)

Display This Question:

If = Q18 = (1)

Q23 **Quand un vaccin COVID-19 est disponible pour vous, le prendrez-vous ?**

- Oui (1)
- Non (2)
- Ne sait pas (3)

Display This Question:

If = Q18 = (1)

Q24 **Veuillez indiquer dans quelle mesure vous êtes honnêtement d'accord ou en désaccord avec l'énoncé : « Quand un vaccin COVID-19 me sera personnellement offert, je le prendrai »**

- Tout à fait en désaccord (Absolument pas) (1)
- Plutôt en désaccord (probablement pas) (2)
- Indécis (Ni d’accord ni en désaccord) (3)
- Plutôt d'accord (probable) (4)
- Tout à fait d'accord (Absolument) (5)

Display This Question:

If = Q18 = (2), (3)

OR = Q24 = (1), (2), (3)

Q25 **Veuillez sélectionner les raisons pour lesquelles vous refusez la vaccination lorsqu'elle sera disponible.** *(vous pouvez choisir plus d'une option)*

- Le vaccin n’est pas sûr ou est dangereux (1)
- Je n'ai pas assez d'informations sur le vaccin (2)
- J'éprouverai des effets secondaires et je tomberai malade à cause du vaccin (3)
- Je n'en ai pas besoin (4)
- Le centre de vaccination est trop loin (5)
- Le vaccin n’est pas efficace (6)
- La COVID-19 n'est pas assez grave pour nécessiter un vaccin (7)
- Je ne fais pas confiance au gouvernement ou à la prise en charge en cas d’effets indésirables graves (8)
- Je serai allergique au vaccin (9)
- Autre (10)

Display This Question:

If = Q25 = (1)

Q26 **Vous avez sélectionné: «Le vaccin n’est pas sûr ou est dangereux». Pourriez-vous expliquer plus en détail pourquoi vous pensez que le vaccin n’est pas sûr ou dangereux.**

**________________________________________________________________**

Display This Question:

If = Q25 = (10)

Q27 **Vous avez sélectionné «Autre». Veuillez fournir plus d'informations.**

_______________________________________________________________

Display This Question:

If = Q18 = (1)

Q28 **Combien de temps attendrez-vous pour recevoir un vaccin COVID-19 ?**

- Je ne prendrai pas le vaccin COVID-19 (1)
- j'attendrais plus d'un an (2)
- j'attendrais entre 6 et 12 mois (3)
- j'attendrais au moins 6 mois (4)
- Dès qu'il est disponible pour moi (5)

Display This Question:

If = Q18 = (1)

Q29 **Si vous deviez recevoir un vaccin COVID-19, à quel point pensez-vous que le processus global serait facile ou difficile ? (par exemple, prise de rendez-vous, se rendre au site de vaccination).**

- Très difficile (1)
- Un peu difficile (2)
- Ni facile ni difficile (3)
- Légèrement facile (4)
- Très facile (5)

Display This Question:

If = Oui to **Avez-vous des enfants de moins de 18 ans ? (Q12)**

Q30Le vaccin COVID-19 est déjà approuvé pour les enfants âgés de 15 ans et plus, et pourrait à l'avenir être approuvé pour les enfants plus jeunes. Dans quelle mesure êtes-vous d'accord avec cette affirmation :

**« Je souhaite que mon ou mes enfants (moins de 18 ans) reçoivent le vaccin COVID-19 lorsqu'il leur est proposé »**

- Pas du tout d'accord (1)
- Plutôt en désaccord (2)
- Ni d'accord ni en désaccord (3)
- Plutôt d'accord (4)
- Tout à fait d'accord (5)

**Toutes les personnes**

Q31 Veuillez indiquer dans quelle mesure vous êtes honnêtement d'accord ou en désaccord avec l'énoncé :

**« Je fais confiance à l'innocuité du vaccin COVID-19. »**

- Pas du tout d'accord (1)
- Plutôt en désaccord (2)
- Ni d'accord ni en désaccord (3)
- Plutôt d'accord (4)
- Tout à fait d'accord (5)

Q32 Veuillez indiquer dans quelle mesure vous êtes honnêtement d'accord ou en désaccord avec l'énoncé :

**« Je souhaite que ma famille proche et mes amis prennent le vaccin COVID-19 lorsqu'il sera disponible »**

- Pas du tout d'accord (1)
- Plutôt en désaccord (2)
- Ni d'accord ni en désaccord (3)
- Plutôt d'accord (4)
- Tout à fait d'accord (5)

Q33 Veuillez indiquer dans quelle mesure vous êtes honnêtement d'accord ou en désaccord avec l'énoncé :

**« Le vaccin sera efficace pour prévenir la maladie du COVID-19 »**

- Pas du tout d'accord (1)
- Plutôt en désaccord (2)
- Ni d'accord ni en désaccord (3)
- Plutôt d'accord (4)
- Tout à fait d'accord (5)

Display This Question:

If = Q18 = (1)

Q34 Veuillez indiquer dans quelle mesure vous êtes honnêtement d'accord ou en désaccord avec l'énoncé :

**« Je crains que mon église ou mon groupe religieux ne me permette pas de recevoir le vaccin COVID-19 »**

- Pas du tout d'accord (1)
- Plutôt en désaccord (2)
- Ni d'accord ni en désaccord (3)
- Plutôt d'accord (4)
- Tout à fait d'accord (5)

Display This Question:

If = Q18 = (1)

Q35 Veuillez indiquer dans quelle mesure vous êtes honnêtement d'accord ou en désaccord avec l'énoncé :

**« Je suis préoccupé par les effets secondaires du vaccin COVID-19 »**

- Pas du tout d'accord (1)
- Plutôt en désaccord (2)
- Ni d'accord ni en désaccord (3)
- Plutôt d'accord (4)
- Tout à fait d'accord (5)

Q36 **Connaissez-vous personnellement d'autres personnes qui ont reçu au moins une dose d'un vaccin COVID-19 ?**

- Non (1)
- Oui, une ou deux personnes (2)
- Oui, quelques personnes (par exemple, 3 ou plus) (3)

**Sources of COVID-19-related Information**

Q37 **Quelles sources utilisez-vous généralement pour recevoir de nouvelles informations sur le COVID-19 et les vaccins ?** *(vous pouvez en choisir plusieurs):*

- Facebook (1)
- Whatsapp (2)
- Twitter (3)
- YouTube (4)
- les médias de masse (par exemple, télévision, journaux, radio) (5)
- Internet (par exemple, Google, Wikipedia, sites Web d'actualités en ligne) (6)
- Ministère de la Santé, de l’Hygiène Publique et de l’Accès Universel aux Soins ou agents de santé (par exemple, médecins, infirmières, pharmaciens) (7)
- Représentants du gouvernement (p. ex., préfets, ministres, commissaires) (8)
- amis ou membres de la famille (9)
- les chefs religieux (10)
- autre (11)
- rien (12)

Q38 Veuillez évaluer votre niveau d'accord en fonction de l'énoncé suivant:

**« Je fais confiance aux nouvelles que je trouve sur les réseaux sociaux »**

- Pas du tout d'accord (1)
- Plutôt pas d'accord (2)
- Ni d'accord ni en désaccord (3)
- Plutôt d'accord (4)
- Tout à fait d'accord (5)

**Oxford AZ**

Q39 **Avez-vous vu, entendu ou lu des informaiton sur le vaccin Oxford AstraZeneca (AZ) récemment ?**

- Oui (1)
- Non (2)
- Pas certain (3)

Display This Question:

If = Q39 = (1)

Q40 **Où avez-vous récemment vu, entendu ou lu des informations sur le vaccin Oxford AstraZeneca ?** *(vous pouvez en choisir plusieurs):*

- Facebook (1)
- Whatsapp (2)
- Twitter (3)
- YouTube (4)
- les médias de masse (par exemple, télévision, journaux, radio) (5)
- Internet (par exemple, Google, Wikipedia, sites Web d'actualités en ligne) (6)
- Ministère de la Santé, de l’Hygiène Publique et de l’Accès Universel aux Soins ou agents de santé (par exemple, médecins, infirmières, pharmaciens) (7)
- Représentants du gouvernement (p. ex., préfets, ministres, commissaires) (8)
- amis ou membres de la famille (9)
- les chefs religieux (10)
- autre (11)

Display This Question:

If = Q39 = (1)

Q41 **En général, comment évalueriez-vous les nouvelles que vous lisez sur le vaccin Oxford AstraZeneca ?**

- Extrêmement négatif (1)
- Un peu négatif (2)
- Ni positif ni négatif (3)
- Un peu positif (4)
- Extrêmement positif (5)

Q42 **Avez-vous lu des histoires récemment sur** **l'indécision entourant l’utilisation du vaccin Oxford AstraZeneca en Europe et en Amérique du Nord ?**

- Oui (1)
- Non (2)
- Pas certain (3)

Display This Question:

If = Q39 = (1)

AND = Q18 = (1)

Q43 **Veuillez évaluer votre niveau d'accord en fonction de l'énoncé suivant:**

«  **L'indécision entourant le déploiement/ l’utilisation du vaccin Oxford AstraZeneca en Europe et en Amérique du Nord m'a rendu moins certain d'accepter le vaccin contre la COVID-19. »**

- Pas du tout d'accord (1)
- Plutôt pas d'accord (2)
- Ni d'accord ni en désaccord (3)
- Plutôt d'accord (4)
- Tout à fait d'accord (5)

**Omicron**

Q44 **Avez-vous entendu parler d'Omicron - la nouvelle variante COVID-19 ?**

- Oui (1)
- Non (2)
- Pas certain (3)

Display This Question:

If = Q44 = (1)

Q45 Veuillez évaluer votre niveau d'accord en fonction de l'énoncé suivant:

**« La nouvelle variante du COVID-19, Omicron, est dangereuse. »**

- Pas du tout d'accord (1)
- Plutôt pas d'accord (2)
- Ni d'accord ni en désaccord (3)
- Plutôt d'accord (4)
- Tout à fait d'accord (5)

Display This Question:

If = Q44 = (1)

Q46 Veuillez évaluer votre niveau d'accord en fonction de l'énoncé suivant:

**"La nouvelle variante du COVID-19, Omicron, me fait peur"**

- Pas du tout d'accord (1)
- Plutôt pas d'accord (2)
- Ni d'accord ni en désaccord (3)
- Plutôt d'accord (4)
- Tout à fait d'accord (5)

**COVID-19 knowledge**

Q47 **À votre connaissance, COVID-19 est ...:** *(veuillez sélectionner votre réponse dans chaque liste déroulante)*

|  |  |
| --- | --- |
| une maladie grave transmise entre les personnes (1) | ▼ Oui (1), Non (2) Je ne sais pas (3) |
| une maladie grave transmise aux humains par des animaux sauvages (9) | ▼ Oui (1), Non (2) Je ne sais pas (3) |
| une arme biologique conçue par le gouvernement chinois (2) | ▼ Oui (1), Non (2) Je ne sais pas (3) |
| un virus conçu par l'industrie pharmaceutique pour vendre ses médicaments (3) | ▼ Oui (1), Non (2) Je ne sais pas (3) |
| une exagération de la part des médias d'information pour provoquer la peur et la panique (4) | ▼ Oui (1), Non (2) Je ne sais pas (3) |
| un fléau causé par les péchés et l'incrédulité des êtres humains (5) | ▼ Oui (1), Non (2) Je ne sais pas (3) |
| conçu pour réduire ou contrôler la population (6) | ▼ Oui (1), Non (2) Je ne sais pas (3) |
| une arme biologique conçue par le gouvernement américain (7) | ▼ Oui (1), Non (2) Je ne sais pas (3) |
| dû à l'installation de la technologie 5G dans le pays (8) | ▼ Oui (1), Non (2) Je ne sais pas (3) |

Q48 **Selon vous, quels médicaments sont efficaces pour traiter la COVID-19 ? (Si vous ne savez pas, veuillez ignorer cette question)**

________________________

**Your Feelings - 1**

Q49 Dans quelle mesure appliquez-vous chacun des comportements suivants par mesure de précaution contre la COVID-19? (**Veuillez répondre aussi honnêtement que possible).**


 **Pratiquer une hygiène régulière et se laver les mains**

- Pas du tout (1)
- Rarement (2)
- Parfois (3)
- Très souvent (4)
- Toujours (5)

Q50 **Désinfecter les surfaces à la maison**

- Pas du tout (1)
- Rarement (2)
- parfois (3)
- Très souvent (4)
- Toujours (5)

Q51 **Porter un masque sur ma bouche et mon nez dans les lieux publics**

- Pas du tout (1)
- Rarement (2)
- parfois (3)
- Très souvent (4)
- Toujours (5)

Q52 **Utiliser un désinfectant pour les mains ou gel hydroalcoolique**

- Pas du tout (1)
- Rarement (2)
- parfois (3)
- Très souvent (4)
- Toujours (5)

Q53 **S'en tenir aux directives de distanciation sociale dans un espace public**

- Pas du tout (1)
- Rarement (2)
- parfois (3)
- Très souvent (4)
- Toujours (5)

**Your Feelings – 2**

Q54 Veuillez indiquer votre accord ou votre désaccord avec chacune des affirmations suivantes. (Veuillez répondre aussi honnêtement que possible).

**Cela me met mal à l'aise de penser à la COVID-19**

- Pas du tout d'accord (1)
- Plutôt pas d'accord (2)
- Ni d'accord ni en désaccord (3)
- Plutôt d'accord (4)
- Tout à fait d'accord (5)

Q55 **J'ai peur de perdre la vie à cause de la COVID-19**

- Pas du tout d'accord (1)
- Plutôt pas d'accord (2)
- Ni d'accord ni en désaccord (3)
- Plutôt d'accord (4)
- Tout à fait d'accord (5)

Q56 **Lorsque je regarde des nouvelles et des histoires sur la COVID-19 sur les réseaux sociaux, je deviens nerveux ou anxieux**

- Pas du tout d'accord (1)
- Plutôt pas d'accord (2)
- Ni d'accord ni en désaccord (3)
- Plutôt d'accord (4)
- Tout à fait d'accord (5)

Q57 **Je crains que ma famille ou mes amis ne contractent le COVID-19**

- Pas du tout d'accord (1)
- Plutôt pas d'accord (2)
- Ni d'accord ni en désaccord (3)
- Plutôt d'accord (4)
- Tout à fait d'accord (5)

**Your Feelings – 3**

Q58 Veuillez indiquer dans quelle mesure vous êtes honnêtement d'accord ou en désaccord avec l'énoncé :

**« COVID-19 est dangereux pour moi en tant que personne »**

- Pas du tout d'accord (1)
- Plutôt en désaccord (2)
- Ni d'accord ni en désaccord (3)
- Plutôt d'accord (4)
- Tout à fait d'accord (5)

Q59 Veuillez indiquer dans quelle mesure vous êtes honnêtement d'accord ou en désaccord avec l'énoncé :

**« COVID-19 est dangereux pour ma famille »**

- Pas du tout d'accord (1)
- Plutôt en désaccord (2)
- Ni d'accord ni en désaccord (3)
- Plutôt d'accord (4)
- Tout à fait d'accord (5)

Q60 Veuillez indiquer dans quelle mesure vous êtes honnêtement d'accord ou en désaccord avec l'énoncé :

**« Je crois que COVID-19 est réel et une menace pour la santé publique »**

- Pas du tout d'accord (1)
- Plutôt en désaccord (2)
- Ni d'accord ni en désaccord (3)
- Plutôt d'accord (4)
- Tout à fait d'accord (5)

Q61 Veuillez indiquer dans quelle mesure vous êtes honnêtement d'accord ou en désaccord avec l'énoncé :

**« Je ne pense pas que le COVID-19 soit une maladie grave »**

- Pas du tout d'accord (1)
- Plutôt en désaccord (2)
- Ni d'accord ni en désaccord (3)
- Plutôt d'accord (4)
- Tout à fait d'accord (5)

**Your Health**

Q62 **Au cours des deux dernières semaines,** **comment évalueriez-vous votre ...**

 **Santé physique**

- Pauvre (1)
- Passable (2)
- Moyenne (3)
- Bonne (4)
- Excellente (5)

Q63 **La qualité du sommeil**

- Pauvre (1)
- Passable (2)
- Moyenne (3)
- Bonne (4)
- Excellente (5)

Q64 **Niveau de bonheur**

- Pauvre (1)
- Passable (2)
- Moyenne (3)
- Bon (4)
- Excellente (5)

**Government Trust and Satisfaction**

Q65 Veuillez indiquer votre accord ou votre désaccord avec chacun des énoncés suivants. *(Veuillez répondre aussi honnêtement que possible)*

 **J'ai confiance dans la réponse du gouvernement togolais à la pandémie de COVID-19.**

- Pas du tout d'accord (1)
- Plutôt pas d'accord (2)
- Ni d'accord ni en désaccord (3)
- Plutôt d'accord (4)
- Tout à fait d'accord (5)

Q66 Veuillez indiquer votre accord ou votre désaccord avec chacun des énoncés suivants. *(Veuillez répondre aussi honnêtement que possible)*

 **Je suis satisfait de la gestion par le gouvernement togolais de la pandémie de COVID-19.**

- Pas du tout d'accord (1)
- Plutôt pas d'accord (2)
- Ni d'accord ni en désaccord (3)
- Plutôt d'accord (4)
- Tout à fait d'accord (5)

**Competition Entry**

Q67 Si vous avez des informations que vous jugez utiles pour nous de connaître, **veuillez utiliser l'espace ci-dessous pour écrire.**

________________________________________________________________

Q68 Si vous souhaitez participer à un tirage au sort pour gagner **1 des 50 bons d'argent mobile (d'une valeur de 2500 XOF)**, veuillez saisir votre adresse e-mail ci-dessous. **Les gagnants du tirage au sort seront sélectionnés en** **janvier 2022.**

Veuillez confirmer votre numéro de téléphone pour recevoir le prix en espèces si vous êtes sélectionné

________________________________________________________________
