# Supplementary material for: COVID-19 vaccine hesitancy and conspiracy beliefs in Togo: Findings from two cross-sectional surveys
Source: PLOS Glob Public Health. 2024 Feb 29;4(2):e0002375. doi: 10.1371/journal.pgph.0002375 (PMC10903826; doi:10.1371/journal.pgph.0002375)

**S1 Fig – Map of Togo showing the regions of the country**

Map sourced from this link - <https://www.nationsonline.org/oneworld/map/togo-administrative-map.htm>


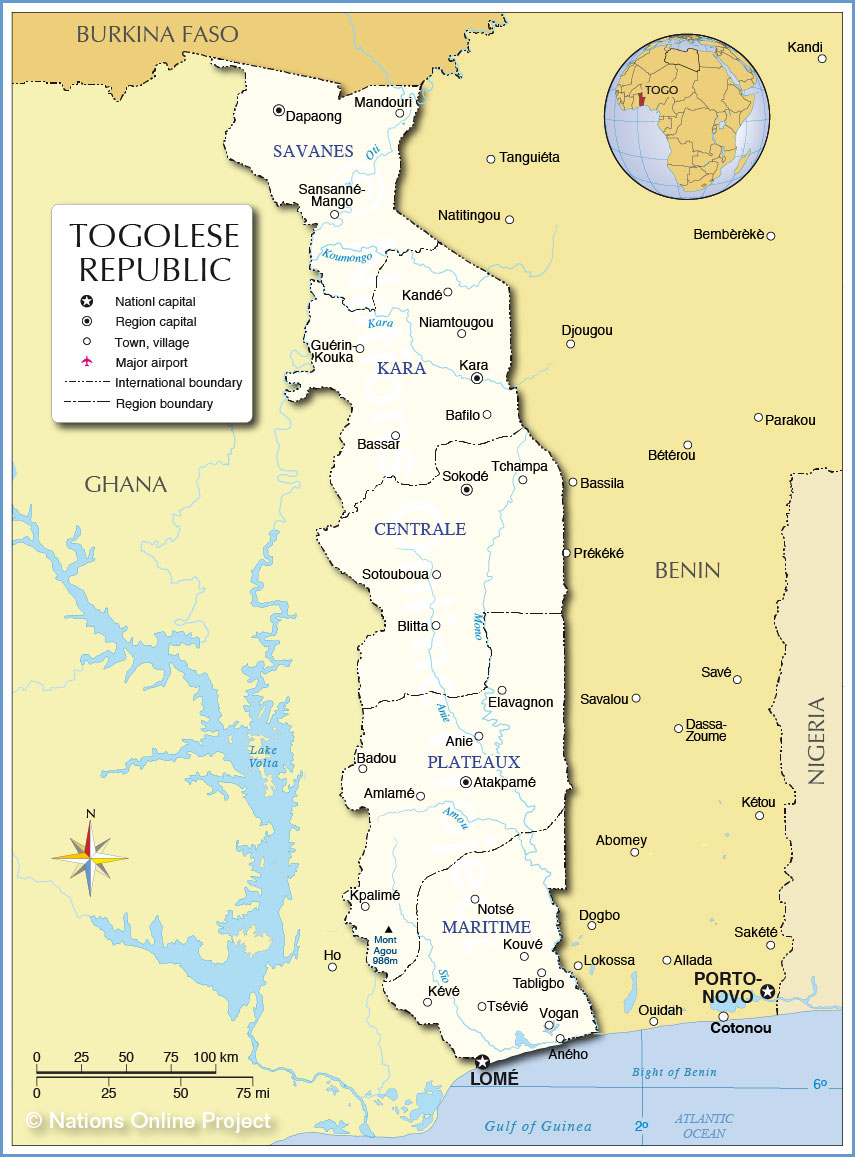

Supplement: S1 Fig — https://www.nationsonline.org/oneworld/map/togo-administrative-map.htm. (DOCX) [file pgph.0002375.s003.docx]
