# Supplementary material for: COVID-19 vaccine hesitancy and conspiracy beliefs in Togo: Findings from two cross-sectional surveys
Source: PLOS Glob Public Health. 2024 Feb 29;4(2):e0002375. doi: 10.1371/journal.pgph.0002375 (PMC10903826; doi:10.1371/journal.pgph.0002375)
Supplement: Attachment — Submitted filename: Comments for Manuscript ID PGPH-D-23-01633.docx [file pgph.0002375.s004.docx]

**Manuscript ID:** PGPH-D-23-01633

**Paper Title:** COVID-19 vaccine hesitancy and conspiracy beliefs in Togo: Findings from two cross-sectional Surveys

**Reviewers Comment**

**Authors Credential Page**

Line 3…. Ministry of Health, of Public Hygiene, and Universal Health Coverage , Lomé, Togo

**Abstract:**

- Line 1…. . Estimates suggest that only 25% of the Togolese population have received at least one dose of any COVID-19
- Line 13….. Between Surveys 1 and 2, overall hesitancy (33.0% to 58.0% respectively)

**Introduction**

Line 46…The COVID-19 pandemic

Line 47…..an estimated 18.2m

Line 64….. 25% of the Togolese population have 64 received at least one dose of any COVID-19 vaccine (by June 2023).(3)

Line 73….. More recent surveys administered in (SSA) countries.

**Methods**

Design

Discussion

Study limitations

Acknowledgement

Conflict of Interest

Authors’ contributions
