# Supplementary material for: COVID-19 vaccine hesitancy and conspiracy beliefs in Togo: Findings from two cross-sectional surveys
Source: PLOS Glob Public Health. 2024 Feb 29;4(2):e0002375. doi: 10.1371/journal.pgph.0002375 (PMC10903826; doi:10.1371/journal.pgph.0002375)
Supplement: Attachment — Submitted filename: Response to reviewers comments v2.docx [file pgph.0002375.s005.docx]

**Response to reviewers comments**

Reference: PGPH-D-23-01633

Title: COVID-19 vaccine hesitancy and conspiracy beliefs in Togo: Findings from two cross-sectional surveys

We thank all the reviewers and editorial team for their time and constructive feedback. Here are the co-author responses (plain text) to reviewer comments (italics).

**Journal Requirements**

*1. Please provide separate figure files in .tif or .eps format only and remove any figures embedded in your manuscript file. Please also ensure all files are under our size limit of 10MB.*

Both figure files have been uploaded as tif files.

**Reviewer #1:**

*This paper reads very well and is sound in approach and presentation. I enjoyed reading it and the authors should be commended for a great piece of work.*

Many thanks for this comment!

*Minor Revisions:

Methodology: More clarity is required regarding how the participants for each questionnaire were recruited. The questionnaire appears to have been conducted via telephone or via a webpage but it is unclear how participants accessed or became aware of the questionnaire.*

We have added the following text into the methods section

‘Togo cell phone numbers have 8 digits. The first two numbers are specific to each of the telecoms providers within the country. The Togo team generated 6 other random numbers to make up a potential phone number, and these numbers were dialled to make an introduction. Numbers of calls were weighted according to the market share of each cell phone network.’

*Line 288 - Re Limitations of the study. Reference to the sample size of survey 2 not meeting the calculated power number should be made.*

Agreed. We have added the following sentence to the limitations paragraph
“When considering the participants who were unvaccinated in Survey 2, we fell short of the required sample size.”

***Reviewer #2****: This study conducted an analysis of vaccine hesitancy among citizens of Togo, utilizing data obtained from two national surveys. The topic holds significance for the epidemiological study of COVID-19. However, there are certain areas that require clarification. This paper can be considered for publication in PLOS Global Public Health after a comprehensive revision.*

Thank you for the review and comments.

*My specific comments are:
1. The purpose and innovations of this paper should be highlighted in the abstract and Introduction. Moreover, please condense the abstract section to include only the most important results and findings.*

We have reduced the number of descriptive results presented in the abstract. Additionally, we have added to one of the introductory sentences to add further context, which now reads as follows -
“Thus, there remains a need to understand reasons for low uptake in countries such as Togo, here focusing on population confidence and trust, essentially characteristics that could potentially be addressed within health promotion strategies .”

We have also added this sentence into the final paragraph within the introduction (line 90)
“Additionally, there is little research more generally focusing around localised beliefs of conspiracy theories and levels of trust, and how this impacts upon vaccine confidence.”
 *2. Page 46-47: Please provide the most recent information on the ongoing COVID-19 pandemic.*

We have updated the Togo figures, which now read
“As of 12 October 2023, the country has reported 39,522 confirmed cases of COVID-19, with 290 confirmed deaths”

And also updated this sentence -
“These numbers are likely to be a significant underestimate, with 7.0 million globally confirmed deaths as of 12 October 2023.”

*3.Page 4, lines 81-84: Please add a reference for this sentence.*

Thank you, we have added a citation to the reference to the Ghana nationwide survey

*4. Page 4, lines 86-89: You mentioned that you’ve already published two studies on vaccine hesitancy in Ghana. Rather than simply changing the location, what are the paper’s innovations?*

As per mention above about adding more context into abstract and introductions, this sentence has been added at this point in the introduction
“Additionally, there is little research more generally focusing around localised beliefs of conspiracy theories and levels of trust, and how this impacts upon vaccine confidence.”

*5. Methods: Design, participants, and procedure: The surveys were conducted between 1-16 December 2020 and 11-28 January 2022. However, researchers may be interested in a more recent survey. Please include the most recent information.*

We carried out two surveys in Togo, at these specific time points. There is no more recent data to refer to in our study.

*6. Methods: There were 1,430 participants in Survey 1 and 212 participants in Survey 2, respectively. Why are the participant numbers so disparate? Would this disparity impact the reliability of the results?*

Yes, in both surveys we considered unvaccinated participants for this specific analysis. We have slightly revised the text to make this clearer, including this within the methods
“However, when considering specifically the participants who were unvaccinated in Survey 2…”
And additionally in the results
“Table 1 presents descriptive statistics of unvaccinated participants from Survey 1 (*N* = 1430) and Survey 2 (*N* = 212).”

And yes, we mention both in the methods, and now additionally in the limitations, about the low sample size for survey 2.

*7. Figures: It is recommended that all the Figures be redrawn due to their lack of clarity.*

We ae unsure about this reviewer comment, as the images look fine to us? Perhaps the submission system reduces resolution of the original image files?
If editorial feedback requires updated images, we will be happy to provide.

*8. Please explain more about how this paper’s findings contribute to the comprehension and prevention of the COVID-19 pandemic.*

We have added into the discussion section (first paragraph, line 260) the following sentence -
“These findings were previously disseminated to Togo health stakeholders for their use in any pandemic-related health promotion or health system strategy.”

We have also added into discussion (lines 278 onwards), this content –

“For example, where specific populations have lower trust in the government but high trust in the health service, the ‘lead messengers’ may best be healthcare workers. Equally, the use of high-profile individuals, who are popular and trusted, could help satisfactorily convey public health messaging. These individuals might include Chiefs, tribal Kings or Queens, or even celebrities. Clearly, there needs to be local strategies that are updated over time to reflect evolving socio-economic dynamics.”

***Reviewer #3:*** *The manuscript is well written and focuses on an important global health problem of acceptability of vaccines by populations to control infectious diseases.*

Thank you for the review and this comment.

*While statistical analysis was done well i want to bring to your attention Table 3. It seems channels of communication was not compared to a baseline. Also, by doing a multivariable statistical analysis, it is important to do a model assessment that selects the best model to use to explain your results.*

Many thanks for your response. Unfortunately, channels of communication were not compared to a baseline since we did not have any baseline data prior to data collection in this cross-sectional study. Rather, instead, we aimed to compare ‘yes’ vs ‘no’ responses for the logistic regression model since this is how channels of COVID-19 information were ultimately collected as part of the survey. This, at least, provided information pertaining to the influences of specific information channels in predicting vaccine hesitancy (e.g., information obtained from the internet).

We also conducted a model assessment as part of model building. We have now explained the process better under ‘Data management and analysis.’ Here, we explain that we initially performed a series of bivariate regressions to ascertain which variables to select for the combined model. The multivariate model consisted of all factors with P values equal to, or smaller than, 0.2 in the bivariate analyses. We also state that assumptions were tested for during model-building.

Thank you for helping us clarify these important details.

*The discussion section did not delve into potential biases from using telephone surveys and how the analysis might have accounted for these biases to strengthen associations observed.*

Many thanks for your comment about potential biases using telephone vs. surveys. We have included the following extra text within the limitation section of the discussion (with an added citation too)

‘Phone coverage in Togo is thought to cover 90% of the population; thus here, our methodology has the potential to access the majority of the country. Our shortfall in reach likely to be in the ‘Last Mile’ populations in hard-to-reach and under-served rural locations, and these are important groups who often lack a voice in healthcare and decision-making. Further studies should consider how best to integrate their feedback into policymaking. There may also be biases in terms of those who agreed to take part versus those who did not answer the calls or did not consent to participation. Sharing of mobile phones among household or communities further introduces potential biases. However, telephone surveys remove the cost of travel and allow access to those spread across a large geographical areas, such as Togo. Therefore, this combined with an inability to conduct face-to-face interviews in the 1st survey due to health concerns and travel restrictions, means that it is the most appropriate method for this research. ‘

*it is not good practice to change a likert scale response to a categorical response because a response of not sure is a standalone response.*

Thanks for this comment. Whilst we understand and agree that this is sometimes the case, we felt that it was justified here because of the definition of, and our operationalization of, vaccine hesitancy. For instance, vaccine hesitancy is defined by the World Health Organization (WHO) as the **delay** **in the acceptance**, or the **blunt refusal of**, vaccines (and we have added this sentence into the introduction, to support our reasoning). This is why we combined ‘no’ (blunt refusal) and ‘I don’t know’ (delay) responses together. This method of assessing vaccine hesitancy has been quite typical in many COVID-19 vaccine hesitancy studies published over the past 3 years. To clarify and support this, we have now included a definition of vaccine hesitancy in the introduction, which was missing before. We have also included a definition of this in the data management and analysis plan in order to justify the coding of hesitancy. Many thanks for helping to clarify this.

***Reviewer #4:***

Thank you for the review.

*The methodology section needs some improvement.
Sample size: Author should justify the chosen sample size, such as effect size. Also, state how the shortfall in survey 2 was addressed.*

Our sample size descriptions are in the methods, see this sentence -
“Prior to data collection, a power analysis was conducted to determine the appropriate sample size. We assumed a confidence level of 95%, and a margin of error of approximately 3-5%, and found that the necessary sample was between 385 and 1067 participants in each survey.”

*Ethical approval: Authors stated ethical approval. However, there is a need to include how patient confidentiality and privacy were ensured during data collection and storage.*

We have added the following into the ‘ethics approval’ paragraph.
“Data storage was electronic, on password-protected devices. Personal identifiers such as participant name or address was not collected.”
There is of course a great deal more content as part of the original ethics submission. If editorial feedback is that we should describe data management processes in more detail, we would be happy to do so.

*Instruments: Authors should expand on the validity and reliability of the measures used, particularly for assessing vaccine hesitancy, governmental mistrust, and conspiracy beliefs. Also, provide details of the psychometric properties of these measures and any prior validation studies conducted in similar contexts*

We have provided citations within the manuscript of the prior usage of these questions, for example in our Ghana surveys. They are fairly standard questions commonly used in vaccine hesitancy studies across the pandemic. We are not aware of any formal validation, so to speak. However, validity for measuring vaccine hesitancy was increased by not only collecting information on whether they felt they would be likely to be vaccinated, but also on whether they had been vaccinated. Additionally, the urgency around the research need (i.e. to support the pandemic response) meant that we used a known bank of questions that have previously provided useful and rapid information.

*Analysis: provide a rationale for covariates in bivariate logistic regression analyses, including potential confounders. Also, explain the process of handling missing data to ensure data quality.*

Many thanks for this comment. We have now better explained our rationale for variable selection in the ‘Data management and analysis’ section. Here, we explain that we initially performed a series of bivariate regressions to ascertain which variables to select for the combined model. The multivariate model consisted of all factors with P values equal to, or smaller than, 0.2 in the bivariate analyses. We also state that assumptions were tested for during model-building, including tests for confounders, multicollinearity, and outliers.

With regards to missing data, we have now explained that any participants who provided missing responses were excluded from the subsequent analyses in order to maintain the quality of the data.

*The author should provide a brief description of conspiracy beliefs to ensure clarity for readers who may not be familiar with them.*

We have added to lines 87-89 in the revised manuscript, with added citation and the following enhanced content -

“For example, a nationwide survey conducted in Ghana found that over 50% of citizens believed, or expressed uncertainty, that COVID-19 was a biological weapon designed by the Chinese government, and that the virus was designed specifically to reduce or control the population. Other widely-circulating conspiracy theories included (incorrectly) associating 5G telenetwork technology in the spread of COVID-19, and that the pandemic was a plague caused by sins and disbelief in human beings.”

*Also, on the association of time and conspiracy belief, the author should provide exact values including confidence intervals can also provide important information about the precision of the estimates.*

As it stands, the results section includes this content, which we think answers this query -
“A Pearson´s Chi-Squared test revealed a significant association between time and conspiracy beliefs, in which the proportion of respondents who indicated agreement increased from 29.2% (CI: 26.6%-31.8%) in Survey 1 (December 2020) to 64.6% (95% CI: 57.9%-71.3%) in Survey 2 (January 2022; χ2 (1) = 103.85, p < .001; Figure 1).”

*Discussion section
251: The result shows the presence of conspiracy theories can impact public perceptions of vaccination. Kindly clarify if the analysis established a causal relationship or if this is an inferred association based on the findings.*

Good point, we have clarified our language to show it is an association. This is the new sentence -
“Our results here show that presence of conspiracy theories are associated with public perceptions of vaccination.”

*The author stated, "the importance of trust in governance and political allegiance in influencing vaccine confidence"(268) Expand on these implications and provide specific recommendations for public health officials on communicating with different population groups and effectively addressing misinformation.
The potential limitations such as underrepresentation (282) and response bias. Provide a detailed discussion of these limitations and suggestions for future research to overcome these challenges. Additionally, discuss any other potential study design limitations or data collection methods.*

As per above reviewer comments, we have enhanced the limitations paragraph with further mention of the small sample size in survey 2, and there is content describing under-representation and response bias. There is additional text around mobile phone coverage , this new content -
“Phone coverage in Togo is thought to cover 90% of the population; thus here, our methodology has the potential to access the majority of the country. Our shortfall in reach likely to be in the ‘Last Mile’ populations in hard-to-reach and under-served rural locations, and these are important groups who often lack a voice in healthcare and decision-making. Further studies should consider how best to integrate their feedback into policymaking.”

Similarly, we have enhanced the ‘trust in governance…’ paragraph with additional content (e.g. see reviewer 2, point 8), including specific points around their implications with integration into health promotion strategies.

***Reviewer #5:***

Thank you for the review.

*Regarding statistical analysis both bivariate and multivariate (COR and AOR) should be computed to identify factors associated your independent variable.*

Many thanks for your response. Indeed, we did compute bivariate and multivariate models to identify relevant factors as part of model building. We have now explained our process better under ‘Data management and analysis.’ Here, we explain that we initially performed a series of bivariate regressions to ascertain which variables to select for the combined model. The final multivariate model consisted of all factors with P values equal to, or smaller than, 0.2 in the bivariate analyses. We also state that assumptions were tested for during model-building.

Thank you very much for helping us to clarify these important details.

*The inclusion and exclusion criteria should clearly stated*

We have now added this, into the first part of the methods (design, participant and procedure). This is the added text-
“Inclusion criteria included those resident in Togo, aged 18 years or over, who had capacity and were capable of providing informed consent. Exclusion criteria included those who were not resident in Togo, unable to provide informed consent, or were aged under 18 years.”

*What do you think the importance of conducting dual cross sectional study?*

We refer to this a little at the start of the strengths and limitations paragraph in the discussion, but have enhanced it, to now read –
“One of the strengths of our study is the relatively large number of respondents, and that it covers two time points, rather than a one-off snapshot of public opinion. These time points also covered pre- and post- the introduction of the COVID-19 vaccines, so thus can give some indication around ‘before and after’ trends.”

*All required files should be presented in form of supplementary material*

Supplementary files were attached to the submission, but unsure if these are made available to reviewers? If editorial feedback requires more content via supplementary information, then we would be happy to provide it.

***Reviewer #6:*** *interesting manuscript*

Thank you for the review, and this comment!

*however, there are some things that deserve commentary
in the intro the assertion that "There are likely to be many factors contributing to the lower mortality in West Africa, including a younger population, better preparedness and better management of outbreaks" is in my opinion unsustainable as the diagnostic data and statistics available in the region are poor to make such an observation.*

This is taking the sentence a little out of context, given the immediately-preceding content, which reads as follows –

“There is evidence showing extensive under-reporting from sub-Sahara Africa (SSA) countries amid a likely higher age-adjusted infection fatality rate (4), and also worsened mortality among hospitalised patients (5). However, it would still appear that most countries on the African continent did not experience high overall death rates observed in high-income settings such as the United Kingdom or United States of America. There are likely to be many contributory factors to the lower mortality in West Africa, including a younger population, improved preparedness (6), and better outbreak management (7).”

The statements are all reinforced with citations, so thus we would respectfully disagree with this reviewers suggestion the statement is unsustainable. If editorial feedback requires changes or clarifications, we’d be happy to consider them.

*it seems fundamental to me to detail the access to telephone services in the country, given that it is described as a poor country and with an important lack of resources, answering a telephone survey may have a marked bias of those who have the capacity to participate in the study.*

As per reviewer 4, we have added content into the limitations paragraph in the discussion, covering phone coverage (90%) and populations we may not be able to reach (rural hard to reach populations), and recommendations for research to actively look to include these forgotten populations in research and decision-making.

*it is not clear why the calculation of sample sizes was so different between the two surveys, requires clarification of the wording.*

A point also raised by other reviewers, we have now made it clearer in the methods and also results that for this analyses, we are interested in unvaccinated (against covid19) groups. In survey 2, this was after the start of the covid19 vaccine rollout.  *the results are expressed in the text and in the tables, which is redundant.*

A previous reviewer asked for more detail on the results, such as confidence intervals!
We have selected key results to describe within the text, and attempted to strike a balance between presentation within text and tables. If editorial feedback would prefer adjustments, then we’d be happy to work on that.
 *the results according to geographical regions are difficult to understand for the reader not familiar with Togo, a map could be attached or characteristics could be given to make this geographical classification relevant.*

Good point, we have added a map of Togo into the supplementary text (S5), and referenced it in the manuscript, in the methods section.

***Reviewer #7:***

Thank you for the review.

*SECTION Abstract:
Page 2 Line 23 - The author states in the first line of the abstract "Estimates suggest that ...." - What are these estimates? Can this source be cited? For example, WHO estimates or whatever study suggested those estimates- please include the reference of these estimates.*

We think PLOS doesn’t permit citations within the abstract; however, we have revised the text to now read -
“Estimates collated at Our World in Data suggest that…”
If editorial feedback requires further edits, then we’d be happy to work on that.

*SECTION Introduction:
Page 4 Line 79 - Capitalize COVID - maintain consistency.*

Corrected, thank you

*Page 4 Line 81 - The author mentioned that studies suggest COVID-19 misinformation - Which studies suggest this? Cite these studies.
Page 4 Line 82 to 84 - The author mentioned a nationwide survey in Ghana that said COVID-19 was a biological weapon - cite this nationwide survey and include it in your references.*

Combining these comments, we have now added citations here (also mentioned by other reviewers).

*SECTION Methods:
Page 5 Line 108 - The author mentioned that the unvaccinated in survey 2 fell short of required number. - What are the consequences / limitations of this findings. Include it in your discussion section under limitations.*

As per other reviewer comments also, we have greatly added to the limitations paragraph in the discussion.

*SECTION Discussion:
Page 17 Line 272 - punctuation correction - period and a comma.*

Thank you, we have corrected this.

*SECTION Conclusion:
Page 18 Line 302 - The author mentions the timeline of the survey - December 2020 and January 2022 - please specify the reason for choosing this timeline. And the findings that justify this time frame? Did this timeline cause any bias in the study results? Mention the justification in the methods as well, and if there are any bias - mention that in the limitations or how you adjusted for it.*

We have added into the methods this sentence -
“Thus, the viewpoints described in this research cover the important timepoints of pre- and post- the pandemic-related vaccine rollout.”

Descriptions of the impact of time are described in the results, e.g. see this section -
“A Pearson´s Chi-Squared test revealed a significant association between time and conspiracy beliefs, in which the proportion of respondents increased from 43.5% (CI: 40.9%-46.1%) in Survey 1 (December 2020) to 81.1% (95% CI: 74.4%-87.8%) in Survey 2 (January 2022; χ2 (1) = 104.72, p < .001; Figure 1).”

And we have added this into the limitations (in discussions) -
“These time points also covered pre- and post- the introduction of the COVID-19 vaccines, so thus can give some indication around ‘before and after’ viewpoints. Further time-points would have allowed us to more closely monitor any evolving temporal trends.”

*ADDITIONAL SECTION REQUIRED: Include a section or a paragraph on public health implications or include as a subgroup in conclusion. There is one line in conclusion that states key future research should focus on closing knowledge gaps. What kind of research are you expecting to be conducted based on the results of your survey? How do your results contribute to what kind of future research needs to be done? How would you fill the knowledge gaps - Elaborate on this? What channels would you employ to improve the health literacy rates?*

We have added in the following content into the discussion -
“Our shortfall in reach likely to be in the ‘Last Mile’ populations in hard-to-reach and under-served rural locations, and these are important groups who often lack a voice in healthcare and decision-making. Further studies should consider how best to integrate their feedback into policymaking.”

We have also revised the conclusion to now read -
“Key future research should focus around knowledge gaps created by the pandemic, such as addressing the impact of misinformation upon routine immunisation uptake, repeated cross-sectional surveys to review temporal trends in vaccine confidence, and how best to proactively educate key stakeholders about emerging misinformation in their communities.”

***Reviewer #8:*** 
*All necessary comments regarding the manuscript guide and paper content have been attached for the authors' correction. Most of the comments are related to the use of acronyms , missing words within the manuscript, absence of major headings and lack of clarity on some components of the methodology.
I will suggest the authors include the dates they access grey literatures on their reference lists*

Thank you for the review. We have accessed the attached document, and described here our edits.

We have corrected the affiliation.

Re use of the word ‘COVID-19’ in the abstract, we feel this is appropriate but are happy to edit if editorial feedback indicates we should.

We have added the word ‘respectively’ into the abstract, as indicated.

Introduction, the ‘m’ in this section – “an estimated 18.2 millions excess global deaths “ - referred to millions, not metres. We have written this out in full to avoid any future reader confusion.

We have corrected the full stop around first mention of citation 3.

We have added the full description of ‘sub-Saharan Africa (SSA)’

We have added content around how participants were telephoned. See our response to reviewer 1 / methodology query.

Re ’ Can your findings be discussed under main heading’ – we do have sub-headings in place throughout the results section. If editorial feedback requests any alterations or revisions, we’d be happy to do so.

Re study limitations, as per several other reviewers, we have now greatly enhanced the limitations paragraph.

Re “Declare your conflict of interest. This should appear after Acknowledgement “. Plus following comment around author contributions.

We do this information via the online submission system, and we think this gets automatically added on, should the paper be accepted.
If editorial feedback requires us to add this information manually to the manuscript at this stage, we’d be happy to do so.
