# Supplementary material for: COVID-19 vaccine hesitancy and conspiracy beliefs in Togo: Findings from two cross-sectional surveys
Source: PLOS Glob Public Health. 2024 Feb 29;4(2):e0002375. doi: 10.1371/journal.pgph.0002375 (PMC10903826; doi:10.1371/journal.pgph.0002375)
Supplement: Attachment — Submitted filename: Response to reviewers comments v2.docx [file pgph.0002375.s006.docx]

**Response to reviewers comments**

Reference: PGPH-D-23-01633

Title: COVID-19 vaccine hesitancy and conspiracy beliefs in Togo: Findings from two cross-sectional surveys

We thank all the reviewers and editorial team for their time and constructive feedback. Here are the co-author responses (plain text) to *reviewer comments (italics).*

**Journal Requirements**

*1. Please review your reference list to ensure that it is complete and correct. If you have cited papers that have been retracted, please include the rationale for doing so in the manuscript text, or remove these references and replace them with relevant current references. Any changes to the reference list should be mentioned in the rebuttal letter that accompanies your revised manuscript. If you need to cite a retracted article, indicate the article’s retracted status in the References list and also include a citation and full reference for the retraction notice.*

There are no changes to the references, and no citations that have been retracted. We have also reviewed their inclusion in the manuscript.

*2. Please amend your detailed Financial Disclosure statement. This is published with the article. It must therefore be completed in full sentences and contain the exact wording you wish to be published.*

*a. State the initials, alongside each funding source, of each author to receive each grant.
b. If any authors received a salary from any of your funders, please state which authors and which funders.*

*If you did not receive any funding for this study, please simply state: “The authors received no specific funding for this work.”*

We have revised the financial disclosure statement, to now include this sentence – “Funding was awarded to MGH.”

None of the authors received a salary from the funder for this research.

**Reviewer responses**

We note the reviewers mostly had no further additional comments (thank you to them). This is the one comment that requires a response, from reviewer 7 -

*“Reviewer #7: Thank you for making the edits.*
*One last concern - i am not sure if discussion and conclusion sections should include citations. These sections should be about your conclusion and findings about the strengths and limitations of the your own study and not cite other studies unless in area where you are trying to find similarities in results to previously existing studies which credit/ discredit the current study. Please make sure this is addressed.”*

We have reviewed recent newly-published papers in PLOS Global Public Health, along with the journal guidance for authors. As such, we have not removed the citations from the discussion section. References have been reviewed for their appropriate inclusion, and we have made no changes. If editorial guidance requires us to make edits, we’d be happy to do so.
